# Region-Specific Slowing of Alpha Oscillations is Associated with Visual-Perceptual Abilities in Children Born Very Preterm

**DOI:** 10.3389/fnhum.2013.00791

**Published:** 2013-11-15

**Authors:** Sam M. Doesburg, Alexander Moiseev, Anthony T. Herdman, Urs Ribary, Ruth E. Grunau

**Affiliations:** ^1^Department of Diagnostic Imaging, The Hospital for Sick Children, Toronto, ON, Canada; ^2^Neurosciences & Mental Health Program, Research Institute, The Hospital for Sick Children, Toronto, ON, Canada; ^3^Department of Medical Imaging, University of Toronto, Toronto, ON, Canada; ^4^Department of Psychology, University of Toronto, Toronto, ON, Canada; ^5^Behavioral and Cognitive Neuroscience Institute, Simon Fraser University, Burnaby, BC, Canada; ^6^Department of Audiology and Speech Sciences, The University of British Columbia, Vancouver, BC, Canada; ^7^Department of Psychology, Simon Fraser University, Burnaby, BC, Canada; ^8^Developmental Neurosciences and Child Health, Child and Family Research Institute, Vancouver, BC, Canada; ^9^Department of Pediatrics, The University of British Columbia, Vancouver, BC, Canada

**Keywords:** preterm, magnetoencephalography, neural oscillation, development, resting state, alpha-band, perception, cognition

## Abstract

Children born very preterm (≤32 weeks gestational age) without major intellectual or neurological impairments often express selective deficits in visual-perceptual abilities. The alterations in neurophysiological development underlying these problems, however, remain poorly understood. Recent research has indicated that spontaneous alpha oscillations are slowed in children born very preterm, and that atypical alpha-mediated functional network connectivity may underlie selective developmental difficulties in visual-perceptual ability in this group. The present study provides the first source-resolved analysis of slowing of spontaneous alpha oscillations in very preterm children, indicating alterations in a distributed set of brain regions concentrated in areas of posterior parietal and inferior temporal regions associated with visual perception, as well as prefrontal cortical regions and thalamus. We also uniquely demonstrate that slowing of alpha oscillations is associated with selective difficulties in visual-perceptual ability in very preterm children. These results indicate that region-specific slowing of alpha oscillations contribute to selective developmental difficulties prevalent in this population.

## Introduction

Children born very prematurely, even in the absence of brain injury and when intelligence is broadly normal, often experience selective developmental difficulties including problems with visual-perceptual abilities (Rickards et al., 2001; Grunau et al., 2002; Atkinson and Braddick, 2007). The biological basis of these issues remains poorly understood. MR imaging has identified numerous structural and functional atypicalities in very preterm children, many of which have been associated with problems in cognitive and perceptual development (Hart et al., 2008; Ment et al., 2009; Miller and Ferriero, 2009). An approach to understanding preterm child brain development which has received somewhat less attention is the mapping of neural oscillations using magnetoencephalography (MEG). MEG is a neurophysiological imaging modality that is particularly effective for characterizing the development of functional brain systems due to its unique combination of spatial and temporal resolution (Hari and Salmelin, 1997), and has been successfully employed to image brain activation in specific cortical systems in preterm infants and children (Nevalainen et al., 2008; Frye et al., 2010). Neural oscillations are known to be critical for brain activity and network connectivity supporting cognition and perception (Joliot et al., 1994; Varela et al., 2001; Ward, 2003; Ribary, 2005; Uhlhaas et al., 2009a), and are altered in many clinical populations (Llinás et al., 1999; Schnitzler and Gross, 2005; Uhlhaas et al., 2009a), including those affecting child development (Murias et al., 2007; Mazaheri et al., 2010). Both spontaneous neural oscillations and their test-dependent dynamics develop throughout childhood and infancy (Clarke et al., 2001; Uhlhaas et al., 2009b; Xiang et al., 2009), including during the developmental epoch corresponding to very premature birth (Okumura et al., 2006; Gonzalez et al., 2011), and are relevant for the maturation of functional brain networks (Uhlhaas et al., 2010). Spontaneous cortical oscillations are characterized by a distinct peak in the alpha-band, and progressive increases in the frequency of spontaneous brain oscillations have been identified as a reliable marker of childhood neurodevelopment (John et al., 1980). Such developmental acceleration in alpha-band oscillations can be reliably measured using MEG. For example, maturational increases in the peak frequency of the mu rhythm have been reported in infants and school-age children (Berchicci et al., 2011) and deviations from typical patterns of spontaneous oscillations often indicate learning disabilities or increased risk for neurological disorders (Ahn et al., 1980).

Using MEG, we previously demonstrated that spontaneous alpha oscillations (8–14 Hz) in school-age children born very preterm are slowed (Doesburg et al., 2011a). Atypical spontaneous alpha oscillations were also found to be associated with poor visual-perceptual abilities, and linked to extensive neonatal procedural pain, in children born at extremely low gestational age (Doesburg et al., 2013a). Such persistent alterations in the spectral structure of spontaneous brain oscillations may contribute to life-long cognitive difficulties in this group, as adults born at extremely low birth weight express altered ratio of high-frequency to low-frequency oscillations (Miskovic et al., 2009). Pronounced differences in long-range alpha-band MEG connectivity have also been identified in very preterm children during the performance of a visual short-term memory task, and these alterations were associated with selective visuospatial difficulties in this group (Doesburg et al., 2011b). Together, these findings indicate that spontaneous oscillations are slowed in very preterm children, and cortical alpha-band connectivity dynamics supporting task processing are not typically expressed in this group. Our previous research has indicated that altered alpha oscillations are strongly related to selective difficulties with visual-perceptual abilities in very preterm children, rather than to general intellectual ability (Doesburg et al., 2011b, 2013a).

Previous studies of altered MEG alpha oscillations in very preterm children and their relation to cognitive development have analyzed data at the sensor level (Doesburg et al., 2011a,b, 2013a), which does not reveal the contribution of specific brain regions, thus limiting interpretation of the underlying functional systems involved. In the present study we investigated slowing of alpha oscillations in very preterm children within specific brain regions and examined their relation to difficulties in visual-perceptual abilities. To this end, we recorded spontaneous-eyes-open MEG activity from a group of school-age children born very preterm and full-term control children. Beamformer source analysis was employed to reconstruct brain activity from 72 locations distributed throughout the brain. These locations were predicated on an anatomical brain parcellation scheme in order to estimate activity within multiple functionally distinct brain regions. Peak oscillatory frequency was obtained for each analyzed brain region and compared with neuropsychological assessments.

## Materials and Methods

### Subjects

Groups of 27 very preterm (≤32 weeks GA) children and 27 full-term controls were tested as part of a longitudinal study investigating the long-term impact of neonatal procedural pain on the neurocognitive development of very preterm children (i.e., Grunau et al., 2007, 2009). Full-term control children were recruited either through pediatricians in infancy or from the community at school-age. The groups were matched on age, sex, and handedness. Both groups comprised 17 girls and 8 boys. Children had been excluded if they were diagnosed with a major sensory (hearing, vision), motor, or cognitive impairment, or had periventricular leukomalacia (PVL) or grade III–IV intraventricular hemorrhage (IVH) on neonatal ultrasound according to Papile’s classification (Papile et al., 1978). Following inspection of movement during the MEG scan, one very preterm child, and one full-term control child were excluded due to excessive motion. The resulting group of 26 preterm children (mean age 7.76 years; SD = 0.46 years) consisted of 8 boys and 16 girls; the group of 26 full-term controls (mean age 7.66 years; SD = 0.28 years) was comprised of 9 boys and 17 girls. Both groups contained 24 right handed children and 2 left handed children. The neonatal characteristics of the preterm group are presented in Table 1.

**Table 1:** **Neonatal characteristics of the very preterm group**.

Gestational age (weeks)	29.82 (2.17)
Birth weight (g)*	1358.62 (402.68)
Singleton (# subjects)	18
Early illness severity (SNAP-II)*	10 (11)
Days on mechanical ventilation*	13 (7)
IVH (Grade I–II; # subjects)	2

*One very preterm child was recruited from outside the longitudinal cohort, excluding scores for certain neonatal variables.

### MEG recording

Two minutes of spontaneous-eyes-open MEG activity was recorded using a 151-channel whole-head CTF Omega system (CTF Systems, Coquitlam, BC, Canada). Subjects were supine during recording and were instructed to maintain visual fixation on a “happy face” stimulus which was presented 40 cm above their eyes. A research assistant accompanied each subject within the magnetically shielded room to monitor the subjects. Data were digitized continuously at 1200 Hz, stored offline for analysis, and subsequently downsampled to 600 Hz. Fiducial coils were attached at the nasion as well as the right and left preauricular points, and each was energized at a distinct high narrow-band frequency. T1 weighted volumetric MRI images were also collected (1.5 T). Due to practical limitations imposed by multimodal neuroimaging in special child populations, MRI images were not available for all subjects. For those subjects without usable MRIs, a substitute matching MRI was found using the NIH database. MRIs for 4 very preterm children and 22 typically developing controls were taken from the NIH pediatric database. To obtain matching MRIs for individual subjects several candidate volumetric MRIs were selected from the database based on small differences between MEG and candidate MRI fiducial points. A best-matching structural MRI was then fitted to the subject’s digitized head surface manually.

### MEG analysis

Head motion was corrected for by obtaining a dipole source solution for each fiducial coil 30 times/s throughout the recording of spontaneous activity. The MEG data were then transformed to a common position by performing an inverse solution, data rotation, and forward solution 30 times/s (Wilson et al., 2007). To investigate slowing and reduction in the magnitude of spontaneous alpha oscillations in preterm children, we reconstructed the activity of multiple brain regions. The regions of interest for the source reconstruction were based on an anatomical brain parcellation scheme (see Kötter and Wanke, 2005; Bezgin et al., 2008). This approach was chosen because it provides multiple functionally distinct regions which are distributed throughout the brain, and because this approach was successfully employed by a MEG study of functional brain activity and its relation to cognition, from which the set of source locations used in the present study were obtained (Diaconescu et al., 2011). A list of each source location is provided in Table 2, and a depiction of each seed location in brain space is available in Doesburg et al. (2013b). Each subject’s MRI was warped into a common Talairach space using SPM2. The 72 locations were then warped back into each individual’s brain space.

**Table 2:** **Each source location used in the analysis of spontaneous MEG activity, together with associated Brodmann area and Talairach coordinates**.

Source	BA	Hemisphere	*X*	*Y*	*Z*
Anterior cingulate cortex	32	Midline	0	32	24
Posterior cingulate cortex	23	Midline	0	−32	24
Retrosplenial cingulate cortex	30	Midline	0	−48	12
Subgenual cingulate cortex	25	Midline	0	16	−8
A1 (primary auditory)		Left	−40	−14	4
A2 (secondary auditory)	22	Left	−60	−14	4
Frontal eye fields	6	Left	−36	8	56
Anterior insula	13	Left	−36	16	−4
Claustrum		Left	−36	−8	−4
M1 (primary motor)	4	Left	−24	−24	56
Inferior parietal cortex	40	Left	−44	−48	20
Angular gyrus	39	Left	−44	−64	28
Precuneus	7	Left	−8	−64	54
Superior parietal cortex	7	Left	−28	−56	54
Centrolateral prefrontal cortex	46	Left	−48	32	12
Dorsolateral prefrontal cortex	9	Left	−48	36	32
Dorsomedial prefrontal cortex	8	Left	−8	36	40
Medial prefrontal cortex	10	Left	−8	48	20
Orbitofrontal cortex	11	Left	−24	44	−20
Frontal polar	10	Left	−24	64	4
Ventrolateral prefrontal cortex		Left	−48	32	−8
Parahippocampal cortex		Left	−28	−16	−16
Dorsolateral premotor cortex	6	Left	−28	0	60
Medial premotor cortex	6	Left	−4	0	60
Ventrolateral premotor cortex	9	Left	−44	4	24
Pulvinar		Left	−16	−28	4
S1 (primary somatosensory)	3	Left	−40	−28	64
S2 (secondary somatosensory)	43	Left	−56	−16	16
Middle temporal cortex	21	Left	−64	−24	−12
Inferior temporal cortex	20	Left	−64	−24	−24
Temporal pole	38	Left	−52	12	−28
Superior temporal cortex	22	Left	−52	−4	−8
Ventral temporal cortex		Left	−32	−28	−28
Thalamus (ventral lateral nucleus)		Left	−8	−8	4
V1 (primary visual)		Left	−4	−84	−4
V2 (secondary visual)		Left	−4	−96	8
Cuneus	18	Left	−20	−88	20
Fusiform gyrus	19	Left	−20	−84	−12
A1 (primary auditory)		Right	40	−14	4
A2 (secondary auditory)	22	Right	60	−14	4
Frontal eye fields	6	Right	36	8	56
Anterior insula	13	Right	36	16	−4
Claustrum		Right	36	−8	−4
M1 (primary motor)	4	Right	24	−24	56
Inferior parietal cortex	40	Right	44	−48	20
Angular gyrus	39	Right	44	−64	28
Precuneus	7	Right	8	−64	54
Superior parietal cortex	7	Right	28	−56	54
Centrolateral prefrontal cortex	46	Right	48	32	12
Dorsolateral prefrontal cortex	9	Right	48	36	32
Dorsomedial prefrontal cortex	8	Right	8	36	40
Medial prefrontal cortex	10	Right	8	48	20
Orbitofrontal cortex	11	Right	24	44	−20
Frontal polar	10	Right	24	64	4
Ventrolateral prefrontal cortex		Right	48	32	−8
Parahippocampal cortex		Right	28	−16	−16
Dorsolateral premotor cortex	6	Right	28	0	60
Medial premotor cortex	6	Right	4	0	60
Ventrolateral premotor cortex	9	Right	44	4	24
Pulvinar		Right	16	−28	4
S1 (primary somatosensory)	3	Right	40	−28	64
S2 (secondary somatosensory)	43	Right	56	−16	16
Middle temporal cortex	21	Right	64	−24	−12
Inferior temporal cortex	20	Right	64	−24	−24
Temporal pole	38	Right	52	12	−28
Superior temporal cortex	22	Right	52	−4	−8
Ventral temporal cortex		Right	32	−28	−28
Thalamus (ventral lateral nucleus)		Right	8	−8	4
V1 (primary visual)		Right	4	−84	−4
V2 (secondary visual)		Right	4	−96	8
Cuneus	18	Right	20	−88	20
Fusiform gyrus	19	Right	20	−84	−12


*Table adapted from Diaconescu et al. (2011)*.

To reconstruct the activity from each analyzed source location, a minimum variance beamformer method was employed (Sekihara et al., 2001). This method has been shown to be very effective in estimating activity of a given source while maximally attenuating signal contributions from all other sources, as well as removing ocular and non-ocular artifacts (Cheyne et al., 2006, 2007). Note that on the sensor level volume currents result in mixing of brain signals from various locations. Source space analysis mostly eliminates the effects of volume currents, because the latter are already taken into account in the beamformer solution. Theoretically, minimum variance beamformers assume that correlations between sources are small, which might pose a problem when synchronous activity of different brain regions is expected. Extensive study of this question has shown that in practice the adverse effects of source correlations on the reconstructed power are significant only if correlations are very strong (Sekihara et al., 2002). Moreover, the signal-to-noise ratio (SNR) also needs to be relatively high for the correlations to introduce significant issues, such as SNRs observed in averaged evoked responses in event-related experimental paradigms. More advanced methods which take correlations into account show that low SNRs (∼0.1), even when strong correlations exist (∼0.8), do not alter reconstructed power significantly (Moiseev et al., 2011; Moiseev and Herdman, 2013). In the case of oscillatory resting state activity, correlations are moderate and SNRs are relatively small. It is for these regions that beamformer techniques have been successfully applied to resting state MEG data in recent years, including in functional connectivity analyses (Gross et al., 2001; Brookes et al., 2011; Hillebrand et al., 2012).

Data reconstructed from each of the 72 source locations were then filtered at 1 Hz intervals from 1 to 60 Hz (pass-band = *f*  ± 0.05*f*, where f is the filter frequency), using the eegfilt function from the EEGLAB toolbox, a two-way least-squares FIR filter (Delorme and Makeig, 2004). These methods which has previously been effective for estimating the spectral content of oscillatory signals in M/EEG data (Doesburg et al., 2008, 2011a,b). Power was calculated at each frequency for each data point and sensor, and was subsequently averaged across all time points in the 120 s recording session. Peak oscillatory frequency for each brain region for each subject was defined as the frequency expressing maximum power between 5 and 20 Hz, which included the alpha-band (8–14 Hz), as well as other nearby frequencies. This wider frequency range was selected because peak frequencies of MEG oscillations in specific brain regions in atypically developing children have not been extensively studied. Given this, we did not wish to make strong *a priori* assumptions about frequency content in specific brain regions in very preterm children. Furthermore, slowing of alpha rhythms into the upper theta range has been reported in several other clinical populations (Llinás et al., 1999; Sarnthein et al., 2006; Sarnthein and Jeanmonod, 2007, 2008; Boord et al., 2008). These methods for determining peak oscillatory frequency in MEG data have been previously established (Doesburg et al., 2011a).

To test the hypothesis that peak oscillatory frequency is slowed in very preterm children without statistical challenges imposed by multiple comparisons, peak frequency was averaged across all 72 analyzed regions for each subject to obtain a global measure for each subject. Permutation testing was then used to test for global differences between the very preterm and full-term control groups (see Blair and Karniski, 1993). Permutation tests were one-tailed as the direction these effects were predicted from previous results (Doesburg et al., 2011a). Once global group differences were established we tested whether oscillatory slowing was present in each of the 72 analyzed brain regions. Since our goal at this stage of the analysis was to determine which set of regions contributed to the established global pattern of oscillatory slowing, and type II errors are as misleading as type I errors in determining which brain regions are involved in the global pattern and which are not, these tests were not corrected for multiple comparisons. The rationale for this is that the conservative nature of such corrections would likely underestimate the number of areas expressing atypical alpha oscillations and provide a distorted account of the location and extent of altered brain oscillations in very preterm children. Permutation tests were also used to evaluate regional between-group comparisons.

### Associations between MEG activity and psychometric data

Following MEG recording, all children underwent psychometric assessment using the Wechsler Intelligence Scale for Children, 4th Edn. (WISC-IV; Wechsler, 2003) and the Beery–Buktenica Developmental Test of Visual-Motor Integration, 5th Edn. (Beery et al., 2004), administered by a psychometrician. For the present study, we elected to focus on visual-perceptual abilities as previous studies have indicated that altered alpha oscillations in preterm children are strongly associated with visual-perceptual difficulties (Doesburg et al., 2011b, 2013a). The visual perception subscale of the Beery VMI was chosen as an index of visual-perceptual ability, as it has proven to be a sensitive measure in previous studies (Doesburg et al., 2011b, 2013a). The Motor Coordination and Visual-Motor Integration Subscales of the Beery VMI and Full-Scale IQ (FSIQ) from the WISC-IV were also evaluated in relation to the MEG data in order to assess the specificity of relations between altered alpha oscillations and functional abilities in very preterm children, consistent with methods employed in previous research (Doesburg et al., 2011a).

Correlations were examined between each of these psychometric measures, within both the very preterm and full-term groups, and peak oscillatory frequency for each brain region that exhibited slowing. Statistical analysis of correlations between oscillatory slowing and psychometric data were controlled for multiple comparisons using the False Discovery Rate (FDR; see Storey, 2002). The approach taken to multiple comparisons here differs from that in the regional analysis of oscillatory slowing because correlations among oscillatory brain activity and psychometric measures aim to determine whether such relationships exist, rather than characterize the location and extent of phenomena which have already been established as statistically significant on a global level.

## Results

### Oscillatory slowing in children born very preterm

**Figure 1:**
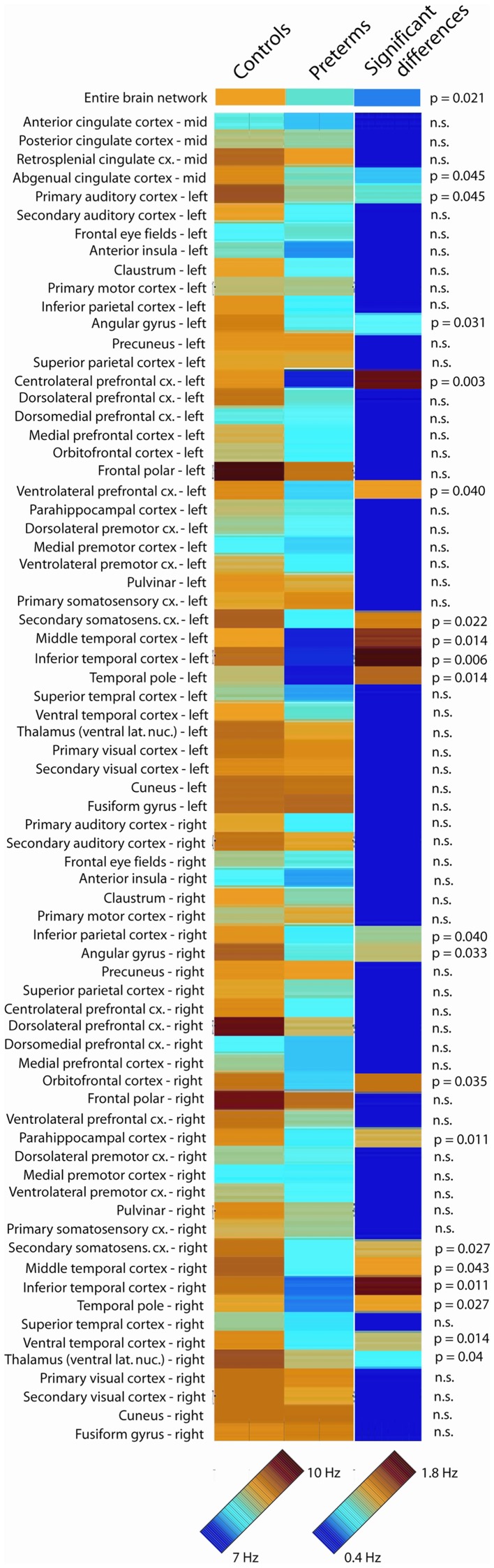
**Oscillatory slowing in very preterm children**. Peak oscillatory frequency in each of the 72 analyzed brain regions for the full-term control and very preterm groups, as well as statistically significant regional slowing in children born very preterm. The 7- to 10-Hz scale bar corresponds to the peak oscillatory frequency for each analyzed region for the very preterm and typically developing groups. The 0.4- to 1.8-Hz scale bar indicates the difference in peak oscillatory frequency between the very preterm and typically developing groups for regions where statistically significant differences were observed (regions for which no significant group differences were observed are presented in dark blue).

Analysis of global peak oscillatory frequency (averaged across all 72 analyzed brain regions) revealed statistically significant slowing in school-age children born very prematurely (*p* = 0.021). Regional analysis of oscillatory slowing revealed that this pattern was widespread and encompassed prefrontal, temporal, and parietal areas, as well as aspects of thalamus and cingulate cortex. Complete results of the analysis of regional oscillatory slowing in very preterm children are presented in Figure 1. To summarize, slowing was widespread in bilateral temporal cortex, including bilateral in temporal areas implicated in the ventral visual processing stream (i.e., ventral and inferior temporal cortex). Parietal regions associated with visual processing were also implicated (i.e., bilateral inferior parietal cortex). Slowing was also present in secondary somatosensory cortex in both hemispheres, and also encompassed numerous in prefrontal regions (i.e., centrolateral, dorsolateral, ventrolateral, and orbitofrontal cortex).

### Oscillatory slowing associated with reduced visual-perceptual ability in very preterm children

Visual-perceptual ability, indexed by the visual perception subscale of the Beery VMI, was associated with oscillatory slowing in multiple regions, including several known to be involved in the processing of visual information including inferior parietal cortex, ventral temporal cortex, and thalamus. Regional slowing was not significantly associated with FSIQ or the Motor or Visual-Motor scores on the Beery VMI for the very preterm children. Peak oscillatory frequency was not associated with any of the psychometric tests for full-term children for any region. Complete results of the analysis of associations between regional oscillatory slowing and visual-perceptual ability in preterm children are provided in Table 3.

**Table 3:** **Associations between slowing of peak oscillatory frequency and visual-perceptual ability, indexed by the visual perception subscore of the Beery VMI, in children born very preterm**.

Source	Correlation coefficient	*P* value (FDR corrected)
Subgenual cingulate cortex (midline)	**0.44**	**0.028**
Primary auditory cortex (left)	n.s.	n.s.
Angular gyrus (left)	**0.53**	**0.019**
Centrolateral prefrontal cortex (left)	n.s.	n.s.
Ventrolateral prefrontal cortex (left)	n.s.	n.s.
Secondary somatosensory cortex (left)	n.s.	n.s.
Middle temporal cortex (left)	n.s.	n.s.
Inferior temporal cortex (left)	n.s.	n.s.
Temporal pole (left)	n.s.	n.s.
Inferior parietal cortex (right)	**0.44**	**0.037**
Angular gyrus (right)	n.s.	n.s.
Orbitofrontal cortex (right)	**0.44**	**0.031**
Parahippocampal cortex (right)	**0.47**	**0.038**
Secondary somatosensory cortex (right)	n.s.	n.s.
Middle temporal cortex (right)	n.s.	n.s.
Inferior temporal cortex (right)	n.s.	n.s.
Temporal pole (right)	n.s.	n.s.
Ventral temporal cortex (right)	**0.54**	**0.035**
Thalamus (right ventral lateral nucleus)	**0.45**	**0.038**

*Significant correlations are presented in bold*.

## Discussion

We provide the first source-resolved analysis of slowing of alpha oscillations in children born very preterm. Identification of the specific brain regions across thalamocortical systems involved in slowed alpha oscillations in very preterm children represents an important step, as altered alpha oscillations may mediate relations between neonatal procedural pain and cognitive outcome in children born at extremely low gestational age (Doesburg et al., 2013a), and alpha oscillations are particularly relevant for altered network connectivity underlying problems in visual-perceptual abilities in very preterm children (Doesburg et al., 2011b). We observed oscillatory slowing within multiple cortical regions encompassing bilateral temporal, parietal, and prefrontal cortical areas. Many regions expressing oscillatory slowing in preterm children are important for visual processing, including regions involved in both the dorsal visual system (i.e., inferior parietal cortex), and the ventral visual system (i.e., inferior temporal cortex, ventral temporal cortex). Slowing of alpha oscillations in preterm children was also prevalent in areas of prefrontal cortex involved in executive function (i.e., dorsolateral prefrontal cortex and orbitofrontal cortex).

The present study provides the first evidence that slowing of peak oscillatory frequency is associated with cognitive outcome in very preterm children. Oscillatory slowing was found to be selectively related to visual-perceptual ability, an area of selective developmental vulnerability in very preterm children (Rickards et al., 2001; Grunau et al., 2002; Taylor et al., 2004), and not associated with overall intellectual function, indexed by FSIQ. These results confirmed associations between slowing of alpha oscillations and visual-perceptual ability were typically, but not exclusively, found in regions involved in visual processing including inferior parietal cortex, angular gyrus, ventral temporal, cortex, and thalamus. There were no associations found between peak oscillatory frequency and neuropsychological ability in the full-term children, indicating that this phenomenon corresponds to neural mechanisms underlying selective developmental difficulties prevalent in very preterm children, rather than normal variance in childhood neurocognitive development.

Although not as prevalent as was observed for visual cortical regions, oscillatory slowing was also present in prefrontal cortical areas (i.e., ventrolateral prefrontal cortex and orbitofrontal cortex), congruent with earlier reports of slowed alpha oscillations over frontal MEG sensors (Doesburg et al., 2011a). This may be significant as executive functions are also an area of selective developmental difficulty prevalent in children born very prematurely (Anderson et al., 2004; Marlow et al., 2007; Mulder et al., 2009). Together with our results implicating region-specific slowing of alpha oscillations with selective difficulties in visual-perceptual abilities, the presence of atypical alpha rhythmicity in prefrontal brain regions in very preterm children suggests that region-specific slowing may be associated with developmental difficulties in executive function as well. Stated more generally, alpha slowing within a specific cortical region may be associated with reduced function in the corresponding psychological domain. In this view, specific cortical systems situated in posterior parietal, inferior temporal, and prefrontal regions may be selectively vulnerable to aspects of adverse neonatal experience, impacting the development of brain oscillations underlying the maturation of cognitive and perceptual functions associated with these areas (Benasich et al., 2008; Uhlhaas et al., 2009b, 2010) and causing selective developmental difficulties in the corresponding domains of executive function and visual-perceptual ability.

The perspective that oscillatory slowing is associated with impairment of normal brain function is consistent with observations of MEG slowing in association with neurological insults in other contexts. For example, regional analysis of excessive low-frequency oscillations has been introduced as a method for mapping abnormal functional brain activity following mild traumatic brain injury (Huang et al., 2012), and the anatomical focus of such excessive slow wave activity appears related to the location of white matter injury, measured using diffusion tensor imaging, in this population (Huang et al., 2009). Furthermore, slowing of peak alpha oscillatory frequency toward the theta range in resting state MEG recordings has been reported in diverse pathological conditions including Parkinson’s disease, neurogenic pain, tinnitus, and major depression (Llinás et al., 1999). Subsequent combination of methods for the analysis of oscillatory slowing with MEG source analysis techniques have indicated that neuroanatomical systems involved in slowing depend on the pathological condition in question, and likely correspond to the nature of symptoms expressed (Schulman et al., 2011). This finding is consistent with the results of the present study which demonstrates that region-specific slowing of alpha oscillations in visual cortical regions are associated with visual-perceptual difficulties in children born preterm. Slowing of alpha oscillations in neuropathic pain has been confirmed using scalp EEG (Sarnthein et al., 2006; Boord et al., 2008). Slowing of alpha oscillations toward the theta range has also been reported using intracranial EEG in patients with neuropathic pain and Parkinson’s disease (Sarnthein and Jeanmonod, 2007, 2008). Oscillatory alpha slowing in M/EEG recordings in numerous pathological conditions is thought to result from deafferentation, particularly in thalamocortical systems (Llinás et al., 2005), and it has been demonstrated that the parameters of alpha oscillations are related to white matter properties revealed using diffusion tensor imaging (Valdés-Hernández et al., 2010).

Slowing of alpha oscillations, together with the purported association of this phenomenon with the loss of integrity in structural brain connectivity, raises questions regarding relations between alpha oscillatory slowing and communication in brain networks. In particular, evidence increasingly indicates that coherence of alpha-band oscillations plays a critical role in communication among brain regions supporting cognition and perception (see Palva and Palva, 2007, 2012 for reviews). Recently, it has been reported that alpha-band oscillatory connectivity is reduced in preterm neonates with brain lesions (Tokariev et al., 2012). Alpha-band oscillatory connectivity during cognitive processing has been found to be slowed and reduced, and associated with selective difficulties in visual-perceptual ability, in very preterm children (Doesburg et al., 2011b). Moreover, this reduced alpha-band phase synchronization was observed in concert with increased interhemispheric theta-band synchronization in the very preterm children, suggesting that task-dependent interactions among brain regions may also be slowed. In light of the prevalence of white matter injury and atypical white matter development in this population (see Khwaja and Volpe, 2008; Miller and Ferriero, 2009), this raises the prospect that increased conduction delays among cortical regions may adversely impact the ability to recruit inter-regional oscillatory network coherence to support task performance.

The observation that slowing of alpha-band oscillations in very preterm children occurred in thalamus is also significant as animal research has demonstrated that thalamocortical mechanisms underlie the generation of alpha oscillations, as well as their slowing under pathological conditions (see Hughes and Crunelli, 2005 for review). Moreover, thalamocortical systems undergo critical phases of development during the gestational epoch corresponding to very premature birth (see Kostovic and Judaš, 2010 for review). The slowing of alpha oscillations, mediated by altered thalamocortical interactions, has been proposed to underlie disruptions of function in several neurological and neuropsychiatric populations (Llinás et al., 1999, 2005). Altered connectivity and white matter development are prevalent in children born very preterm (see Miller and Ferriero, 2009 for review), including altered development of structural (Anjari et al., 2007; Dudink et al., 2007) and functional (Smyser et al., 2010) connectivity in thalamocortical systems. The importance of thalamus for understanding alterations in cortical development in preterm children is further underscored by recent findings that reduced thalamic volume is predictive of reduced cortical volume in preterm infants (Ball et al., 2012).

Our results showing significant slowing in deep brain structures such as the thalamus should be interpreted with caution. Reconstructing MEG signals from deep structures is not only difficult, but in many cases impossible because the SNR is too small (see Quraan et al., 2011). It should be noted, however, that in terms of power the alpha-band signal is the strongest in the brain, improving the likelihood of accurate source reconstruction in this frequency range (Attal et al., 2007). Moreover, prior MEG source analysis studies have reported task-dependent changes involving thalamus (i.e., Schnitzler et al., 2009). Further investigation will be needed to definitively determine the reliability of MEG measures of oscillations power from deep structures such as thalamus. Future research will also be required to examine relations between the structural and functional development of thalamocortical systems, disruption of the normative structure of cortical alpha oscillations, and functional outcomes in specific psychological domains in children who were born very prematurely. Involvement of specific regions identified in the source space analysis of oscillatory slowing in very preterm children should also be interpreted with some caution, as correction for multiple comparisons was not performed across all analyzed 72 regions. As such, potential false positives for individual regions may have arisen. The consistency of slowing located in regions involved in dorsal and ventral visual pathways, however, together with prefrontal cortical regions, is highly suggestive that region-specific slowing in brain structures is associated with selective developmental difficulties prevalent in children born very prematurely.

## Conclusion

We demonstrate that slowing of spontaneous alpha oscillations is evident in widespread brain regions in very preterm children. These alterations of alpha oscillations were concentrated in regions involved in visual processing including thalamus, posterior parietal cortex, and interior temporal cortex, as well as in prefrontal regions relevant for executive functions. We provide the first evidence that slowing of spontaneous alpha oscillations is associated with functional outcome in preterm children. This association between slowing of alpha oscillations in particular brain regions and function was specific for visual-perceptual abilities, and was typically found in posterior parietal and inferior temporal brain regions implicated in visual processing. These results indicate that area-specific atypicalities in alpha oscillatory brain activity are associated with selective difficulties in children born very prematurely.

## Conflict of Interest Statement

The authors declare that the research was conducted in the absence of any commercial or financial relationships that could be construed as a potential conflict of interest.
